# SmartChemist—Simplifying Communication About Organic Chemical Structures

**DOI:** 10.1021/acs.jcim.5c00599

**Published:** 2025-06-09

**Authors:** Torben Gutermuth, Patrick Penner, Jochen Sieg, Uschi Dolfus, Matthias Rarey

**Affiliations:** ZBH—Center for Bioinformatics, 14915 University of Hamburg, Albert Einstein Ring 8-10, 22761 Hamburg, Germany

## Abstract

Communication between collaborators of different (scientific) backgrounds can be difficult in any interdisciplinary work. Frequently used language of different disciplines can be partially unknown or sometimes even contradict each other. One example of this is knowledge about organic chemical nomenclature used by chemists. Although highly efficient, this nomenclature can be challenging to learn and confusing for scientists new to it. Today, interdisciplinary teams in chemistry often consist of a diverse range of experts, including those from biology, pharmacology, medicine, computer science, and mathematics. This can complicate communication regarding chemical structures, impeding a productive work environment and potentially introducing errors. To address this issue, we present a new web tool called SmartChemist. This tool enables users to upload molecular files or enter SMILES of molecules. It then displays the names of identified substructures, including cyclic substructures, functional groups, and common biologically relevant organic molecules. It utilizes a database of over 40,000 patterns that have been carefully generated for this purpose, and it only shows the most specific substructures found while enabling novices to inquire about all substructures found.

## Graphical abstract

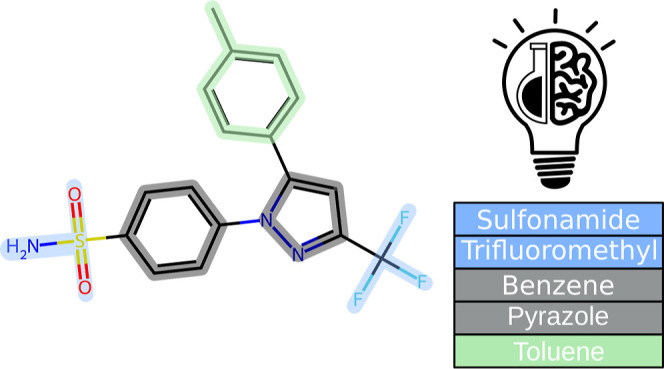

## Introduction

Drug discovery is a collaborative effort that brings together scientists from diverse, often interdisciplinary backgrounds. These professionals work on developing new drugs, which involves discussing compound collection patterns,^1^ brainstorming molecule alterations,^2^ and finding bioisosteric replacements.^3^ Effective communication is essential in this complex, collaborative environment and particularly crucial when discussing substructures of a small molecule. In 2023, the number of approved biological drugs matched that of small molecule drugs for the first time.^4^ So it is not only important to communicate about small molecule drugs, but also about patterns consisting of biologically relevant structures.

In addition to textbooks, many online resources provide names for functional groups, heterocycles, and well-known biological entities. However, these resources typically focus on specific definitions, such as articles about particular functional groups, or offer a limited selection, like the most commonly encountered heterocycles. Using these resources, finding the specific definition a scientist is looking for without knowing its name is, at best, tedious and, at worst, impossible. In particular, names of infrequent monocyclic or bicyclic, or larger, heterocycles are difficult to find without querying the substructures in a chemical database like PubChem.^5^ The time and effort required finding the name of a substructure can be prohibitive for someone trying to verify the correct names of substructures. To address this challenge, we suggest an online resource that allows scientists to execute queries for systematic chemical names directly from the molecules they want to communicate about. Building such a resource requires the names of functional groups, heterocycles, or common biological substructures like sugars, which would have to be available together with a machine-readable molecular representation.

IUPAC is the organization responsible for most of the definitions presented in this publication. They offer the “Blue Book”,^6^ which outlines the official names and rules for constructing IUPAC names of complete organic molecules and substructures. Additionally, they provide the “Gold Book”,^7^ which includes a glossary of IUPAC terminology. Although the Gold Book has a Web site and is provided as a JSON file, neither it nor the Blue Book provides substructure descriptions in standard cheminformatics formats such as SMILES or SMARTS, making it difficult to use directly. Additionally, the IUPAC names defined in the Blue Book are designed to precisely and uniquely describe complete molecules, rather than to facilitate communication about them. Caffeine is a good example, as its IUPAC name 1,3,7-trimethylpurine-2,6-dione—while expertly fulfilling its purpose—does not help scientists less familiar with chemical nomenclature locate the substructures. Without training, it is unclear which part of the name corresponds to which part of the structure and what the different terms describe. In addition, many interesting substructures are not part of the name. Which part is an imidazole ring? Does the molecule’s core and the two carbonyl groups have a trivial name? Which parts of the structure can be described as a lactam? Is a uracil substructure hiding within? Even though open-source projects to convert IUPAC names to SMILES exist,^8^ these do not solve this problem. There needs to be a link between the substructure name and a machine-readable pattern that goes beyond what is used to construct IUPAC names.

Few published machine-readable resources, best using SMILES or SMARTS, exist on functional groups, heterocycles, or biologically relevant molecules and their respective names. For biologically relevant molecules, the extensive ChEBI database is available.^9^ It contains well over 180,000 molecules with respective ChEBI names. Outside of biologically relevant molecules, one of the few well-known machine-readable examples for functional groups and some cyclic structures is the invaluable but comparatively sparse DayLight Web site.^10^ The patterns^11^ in these examples are also designed for many tasks, for example, finding a phenol by matching only three atoms, matching any chiral carbon or rotatable bond. They are not necessarily designed to describe different substructures precisely. Descriptions of substructures are also important in other areas of the field. Explaining black-box predictions can be far more understandable when utilizing human-readable explanations like in the exmol package,^12^ which also includes functionality to find functional groups in molecules. ClassyFire^13^ uses a wide variety of categories with machine-readable descriptions like SMARTS and Markush structures to categorize chemical molecules hierarchically.

In this publication, we present a workflow and a collection of patterns that led to the creation of SmartChemist, a novel web service designed for querying named substructures within molecules. In contrast to existing methods, our focus is on generating a complete catalog of functional groups including the large variety of organic ring structures and biologically relevant substructures. In combination with an easy-to-use web interface, SmartChemist greatly simplifies communication about substructures for practitioners from various backgrounds.

Users can upload one or more molecules, and by clicking on the names of specific substructures, the relevant parts of the molecular structure will be highlighted. To support this web service, we have established a comprehensive public database containing over 40,000 substructures encoded as SMARTS patterns. These patterns include names for functional groups, cyclic structures, and biologically relevant entities.

In the following sections, we will describe the process of collecting these patterns, outline our general approach, and present the resulting service along with several practical examples.

## Methods

SmartChemist is designed to assist scientists when communicating substructures of molecules. Which substructures are interesting depends on the scientist. Chemists, for instance, typically focus on functional groups and the names of ring systems. In contrast, biochemists or synthetic biologists may be more concerned with the segments of molecules that consist of biologically relevant subunits. To reflect this, SmartChemist distinguish three broad categories: functional groups, which are defined as small substructures in molecules; cyclic structures, referring to any slightly substituted (hetero)­cycles; and biologically relevant ones. Note that the biologically relevant group also acts as a collection of patterns that do not fit the previous groups, such as toluene or the cresol isomers. Depending on the scientist’s level of expertise, some patterns may be simple to recognize, while others might be too intricate to recall easily. SmartChemist solves this by employing a pattern hierarchy within each subcategory. SmartChemist’s frontend then shows only the most specific pattern matches as default while hiding less specific ones unless requested otherwise. This enables both novices and experienced users to benefit from SmartChemist.

### Creating SmartChemist’s Pattern Collection

#### Functional Group Patterns

The Blue Book^6^ does contain sections about functional groups and their influence on IUPAC names. However, it only discusses the ones relevant to preferred IUPAC names, e.g., amides, but not lactams. The Gold Book^7^ also contains many definitions for functional groups. However, these are not machine-readable, and there is no concrete list of all functional group definitions. Note that the gold book is not complete. The glossary does not include some trivial names of well-known substructures, such as guanidine groups, trifluoromethyl, or sulfate.

The patterns created for SmartChemist have a special requirement which is considered during construction, apart from adhering to the definition and correctly matching the (sub)­structure. Only the atoms of the pattern need to be matched, even if outside atoms or properties are needed for the definition. Examples of this are the azo pattern (see Figure 1a) or the aldehyde pattern, see Figure 1b. Both consist of two atoms connected by a double bond in which, through recursion, it is stated that some must also be connected to a carbon atom, but said carbon atoms are not part of the pattern.

**Figure 1:**
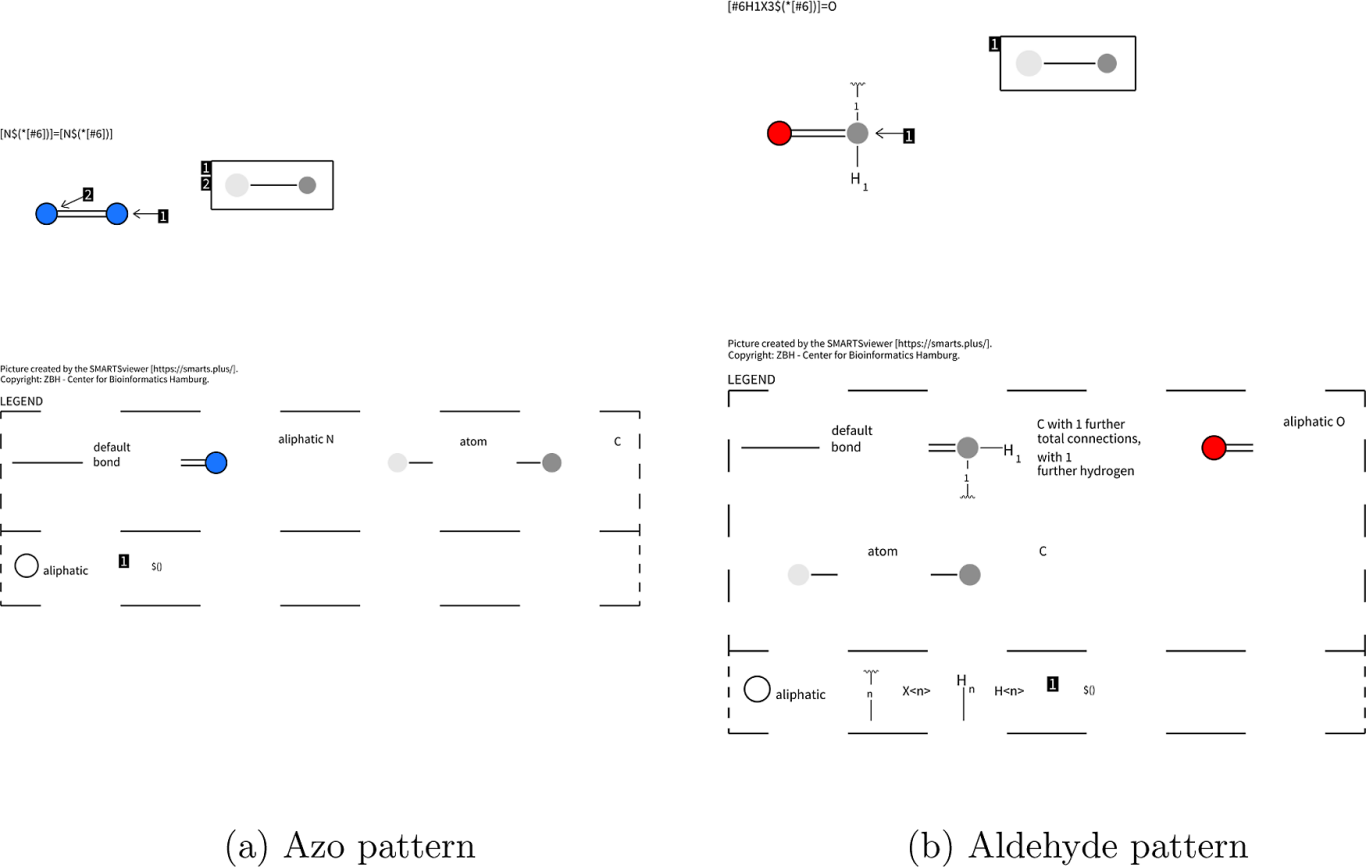
Examples of functional group patterns visualized using SmartsViewer.^14^

While existing resources^10,15^ were utilized as a starting point, all functional group patterns have been manually constructed and improved through an iterative testing phase. All patterns were queried in the Gold Book to double-check both naming and pattern definition. Rarely are there multiple definitions for one name, in which case the one most relevant for drug discovery is chosen. One example is imide, where the first definition of diacyl derivatives of ammonia or primary amines is used. In total, a collection of 158 patterns for functional groups emerged.

#### Cyclic Patterns

The second class of substructures are Ring systems. One possible construction approach is to use the systematic and retained names of IUPAC to construct an IUPAC name for any ring system and use that. However, problems arise when using only IUPAC names, as trivial names are often more “speakable” and frequently used. An example of a trivial name is tetrahydroquinazoline, which corresponds to the IUPAC name 1,2,3,4-tetrahydroquinazoline. As no other tetrahydro variant is reasonable, the trivial name is more straightforward. Another example is the xanthine scaffold found in caffeine, where the IUPAC name, 3,7-dihydropurine-2,6-dione, omits this trivial name. For these reasons, an alternative and less complex approach for collecting patterns was utilized. We utilized all compounds from the PubChem^5^ database and downloaded the SMILES representations and names for every molecule. Using RDKit,^16^ we extracted molecules consisting solely of atoms and bonds within ring systems, while also including exocyclic double bonds to terminal oxygen or sulfur atoms. In addition, we limited the patterns to cyclic structures that could be reasonably used to communicate. Therefore, molecules containing radical electrons, specified isotopes, more than 100 heavy atoms, or mixtures were skipped. If the names found in PubChem contained more than four numbers, ended with “yl”, describing residues of molecules, or began with the identifier “CID”, they were skipped as well. In total, 40,724 different cyclic systems emerged that were used as patterns.

#### Biologically Relevant Patterns

Recognizing and naming simple sugars, specific amino acids, DNA bases, and other frequently occurring molecules in a biological context is another skill only obtained by some. To assist with the communication of biologically relevant substructures, we have added a third category named biological patterns, which consists of 58 manually constructed patterns. These biologicals consist of DNA bases, amino acids, and additional structures such as catecholamine, simple sugars, or common cofactors. The many molecules described in ChEBI^9^ would significantly add to this group, but the number of patterns would prohibitively slow down the application.

### Matching Algorithm

Highlighting all pattern matches found in a molecule quickly becomes overwhelming for larger structures. We considered designing the patterns so that they would automatically not match in some instances. For example, by enforcing that no alcohol is matched in a carboxylic acid or by enforcing the expected number of rings each atom is in within a cyclic system. However, this would lead to further work designing these patterns, and we do believe that these patterns—if annotated correctly—are very helpful for novices in chemical nomenclature to learn. That a purine ring consists of condensed pyrimidine and imidazole rings is trivial to many but helpful to some (see Figure 2c).

**Figure 2:**
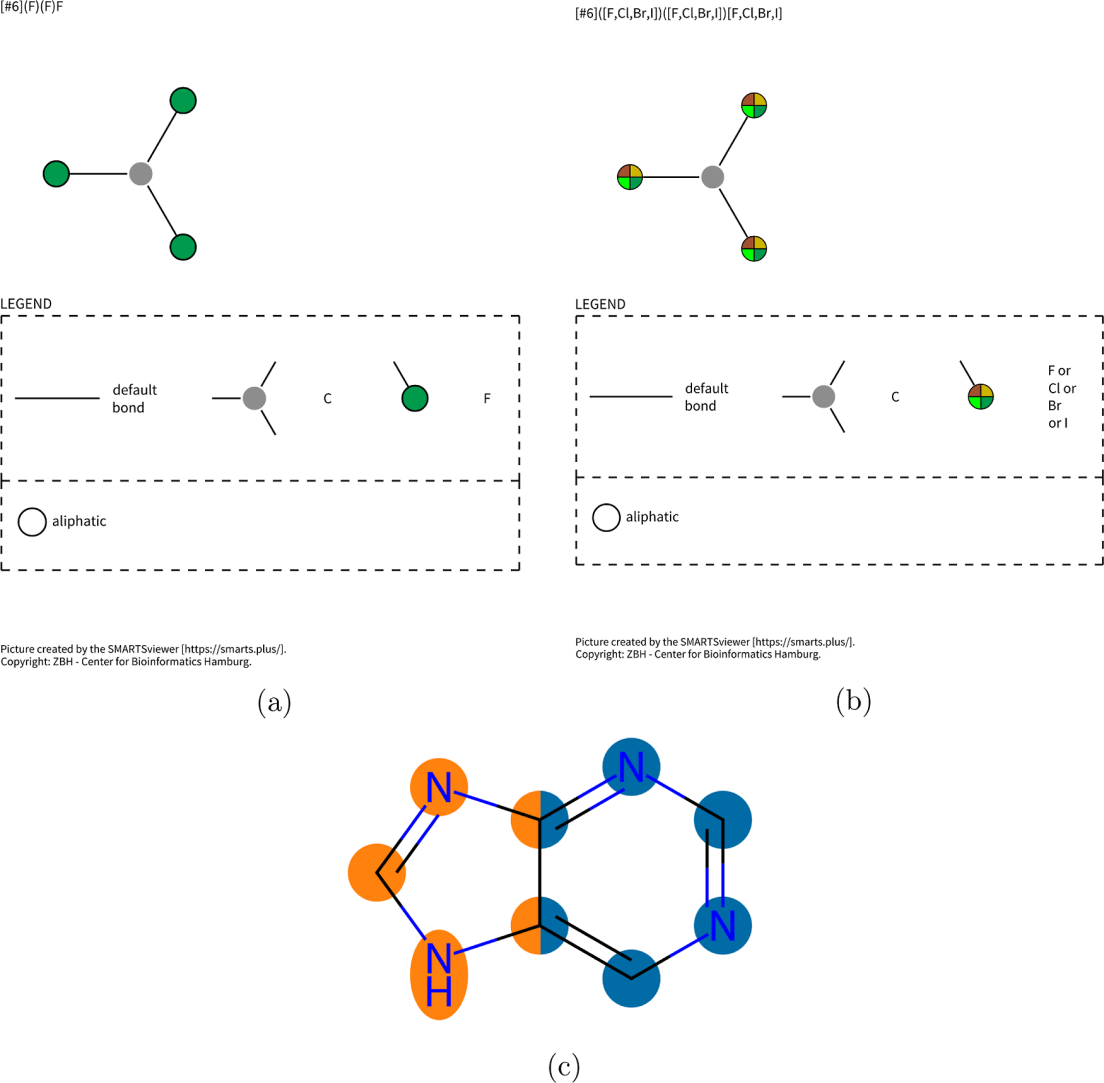
Visualization of the SMARTS for (a) trifluoromethyl and (b) trihalide substructures using SmartsViewer,^14^ and (c) visualization of a purine ring, with the pyrimidine substructure highlighted in blue and the imidazole substructure highlighted in orange.

To formalize this concept, we define the relationship of ’overshadowing’ to pattern matches. A pattern match A is overshadowing pattern match B, if either pattern B is a subset of pattern A and both match or if the atoms and bonds matched by B are a subset of the atoms matched by A. In other words, A describes a larger portion of the molecule or is more specific while always containing the part B would match. As an example for the definition, A could be a carboxylic acid, B the subset alcohol so that any pattern match of B is overshadowed by A.

To not overwhelm users we decided to not display all pattern matches that are overshadowed at first. Upon request, it is possible to display them improving the web service as a resource for novices.

#### Hierarchical SMARTS are Not Enough to Tackle Overshadowing Pattern Matches

SMARTSCompare^17,18^ is a potent tool that can calculate pairs of SMARTS describing subset relationships to each other, which enables us to recognize patterns that are less specific than others. An example is trifluoromethyl groups (see Figure 2a), which describe the same number of atoms as a trihalide (see Figure 2b), with the more specific information on all halogens being fluorine atoms. While a trifluoromethyl group is also a trihalide, no chemist would call it a trihalide, and therefore, trihalide should at least not be shown by default in this case. This is a perfect example of where SMARTSCompare can be used to detect hierarchies and decide on the pattern to be shown. If there are minimal differences between two SMARTS that prevent a subset match, however, this approach fails. It is possible to construct patterns so that no unwanted subset relationships occur in most cases. However, this means that every other pattern has to be considered when designing a new pattern. When, for example, constructing the hydroxy and carboxylic acid patterns ([CX3]­(=O)­[OH,O−]), a slight change in the hydroxy pattern like the addition of the degree ([OHX2, O–H0]) destroys this subset relationship. While trivial to fix in this instance, the problem grows significantly in complexity with every new pattern to consider. Additionally, in the hypothetical case of two patterns not forming a subset relationship but still occasionally matching atom lists with set-subset relationships this approach would also fail and multiple patterns would be shown for the same part of a structure.

Pattern hierarchy allows us to detect more specific patterns in our collection in order to only show the most specific ones. But besides considering the hierarchy of SMARTS, further solutions have to be found to show only the most relevant pattern matches.

#### Overshadowing Mimics Chemical Intuition

Another approach to defining which pattern matches to show by default is to calculate which are overshadowed by others. One pattern, for example, a purine ring, can overshadow another pattern, such as an imidazole ring if the match fully encompasses all atoms and bonds matched by the other pattern (see Figure 2c). If both patterns are of identical size in both atoms and bonds—e.g., the trifluoromethyl/trihalide example—or they contain different atoms, none are marked as overshadowed. But in the first case the more specific one is calculated using SMARTSCompare and therefore hidden. If both pattern matches are of identical size in atoms but not bonds, the one with fewer bonds is marked as overshadowed, which frequently happens when both a bicyclic pattern and a monocyclic pattern match the same atoms with the difference of one bond. An example of this is cyclononane and hydrindane. With the concept of overshadowing, it is trivial to automatically hide monocycles if a bicycle is found to match, hide patterns only matching subsets of functional groups, e .g., hydroxy and carboxylic acid, or hide matching ring systems if larger biological patterns are found.

### Web service Design

#### SmartChemist Workflow

SmartChemist is built on a Django^19^ backend utilizing RDKit.^16^ For task parallelization, Gunicorn,^20^ Redis,^21^ and Celery^22^ are used, and a PostgreSQL database is used for the patterns, queries, and bug reports. It is possible to upload molecules using a SMILES string, even multiple molecules and named ones, or as a SMILES or SD File.

To reduce the number of SMARTS matches, some prefilters are applied checking whether a SMARTS match in the molecule is possible at all. If, for example, a molecule does not contain enough rings or enough atoms of a particular element, then the expensive SMARTS matching step is skipped. Constructed patterns are saved during multimolecule queries to avoid reconstruction. The backend calculates all matching patterns, including their names, matching atoms, groups, and whether they are overshadowed, and provides this information to the front end in JSON Format. The front end is built using Bootstrap and Vanilla Javascript with a custom-built algorithm to highlight what the user has chosen on the fly.

#### Improvement of Existing Patterns in the Future

The SmartChemist pattern collection is an initial release and is open to improvements. We have immediately planned to allow expert users to provide feedback when they encounter inconsistencies, such as incorrect assignments, missing patterns, or spelling errors.

By clicking the “Report problematic/missing pattern” button, users can submit feedback regarding potential issues. When reporting problems, the state of the web server will be saved to a database, which will facilitate the manual reproduction of the problem. We will regularly provide new and improved versions of the SmartChemist pattern collection.

## Results

### Data Set

In summary, the SmartChemist pattern collection consists of 40,724 patterns of cyclic structures, 158 patterns for functional groups, and 58 patterns for biologically relevant molecules and trivial names, resulting in a total of 40,940 patterns. While the cyclic patterns dominate by numbers, the hand crafted functional group and biologically relevant patterns are just as important.

### SmartChemist Web service

To visualize substructures of molecules effectively, we decided only to display a single molecule at once (Figure 3). The molecule’s name is displayed under its visualization if specified in the file or string uploaded to the web service; otherwise, “No Name” is shown.

**Figure 3:**
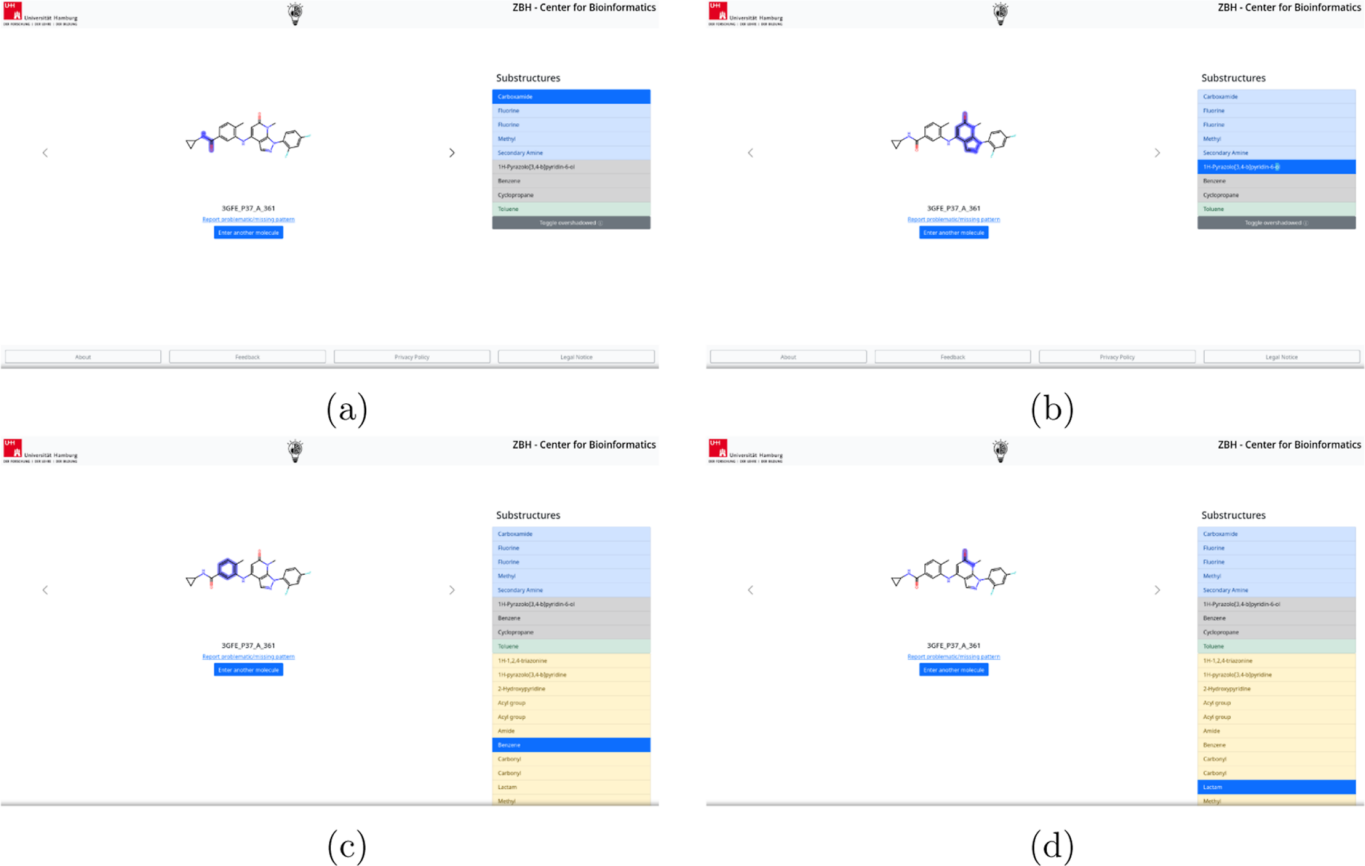
Examples of the Frontend of the SmartChemist web service. The molecule is in the middle; matching patterns are on the right. Pattern names in blue describe functional groups, ones in gray cyclic patterns, ones in green biologically relevant patterns, and ones in yellow overshadowed patterns. Overshadowed patterns are hidden by default. Users can give feedback about patterns by clicking the link under the molecule. Switching the molecule in multimol queries can be done by clicking the arrows.

All names of matching patterns not overshadowed are displayed on the right, with functional groups embedded in blue, cyclic structures embedded in gray, and biologically relevant ones in green. Upon clicking on the name of a pattern, the corresponding part in the molecular graph is highlighted (Figure 3a,b). If one pattern matches multiple times, it is repeated for each match so that new users are not confused by multiple overlapping occurrences.

By clicking the “Toggle Overshadowed” button, users can expand the list of substructures to include all overshadowed patterns, which are displayed in yellow (see Figure 3c through d). Additionally, by clicking “Report Problematic/Missing Pattern” under the molecule name, users can report any issues with the patterns or the web service.

## Discussion

Crafting a database of patterns for communicating about chemical structures is difficult to do perfectly, as there is no single source of truth on how to communicate. IUPAC is the most influential source for the names of substructures and complete molecules, but it would require a significant effort to fully convert the color books into machine-readable definitions. Furthermore, many trivial names exist outside of the scope of IUPAC. In addition, there is no definitive source for trivial names of molecules, even though this problem is solved in practice by resources like PubChem^5^ and ChEBI.^9^ The number of patterns constructed for functional groups and biologically relevant patterns is limited, as they are handcrafted and, therefore, have not reached their maximum potential yet. There will be chemists with higher expertise in functional groups who know further patterns to annotate, find problems in existing SMARTS, or know trivial names that are not part of the existing database.

The generation procedure for cyclic patterns is automated and based on PubChem but contains some heuristics to restrict the number of patterns with limited usability. These criteria are developed based on the first user experiences. Given their heuristic nature, stricter or less strict criteria could improve the overall performance. Our approach aims to balance thoroughness, skipping patterns that would not be used for communication, e.g., too complex names, and including trivial names. There are at least two possible alternatives to this procedure. The first approach would be to go through databases of molecules, e.g., ChEMBL,^23^ find all frequently occurring scaffolds, query their names, and use them as cyclic structures.

While limiting the number of patterns, this approach would fall short of any novel scaffold not yet part of the database. A second approach is to only use the retained names defined by IUPAC and their construction algorithm for names of cyclic structures, ignoring trivial names outside of IUPAC and introducing the problem of implementing the naming algorithm.

Apart from improvements to the database, there are many possible web service improvements. The web service could be extended to feature a molecule editor, give an overview of all molecules like MONA,^24^ showcase identical patterns in multiple molecules, or work not only with SMILES or molecular data but database identifiers like that used in ChEMBL^23^ or ZINC.^25^ The web service could be altered to work like a learning platform, giving queries with either the pattern’s name or the pattern itself, taking the opposite as an answer, or asking students to catch mismatched patterns and names. Even including the patterns in tools like PoseView^26^ or PLIP,^27^ generating an automatic natural language description of interactions between protein and ligand are possible extensions in the future. Lastly, we focused on the English language but support for other languages could greatly enhance the data set.

## Conclusion

We have developed SmartChemist, a novel web service allowing scientists to check how to communicate about substructures in their molecules quickly. It enables novices and experts to brush up on their knowledge of nomenclature and trivial names in an intuitive web front-end. The project’s open-source nature enables any users forbidden or unable to upload molecules of interest to host their own version, for example, within a secluded company network.

The backbone of SmartChemist is the collection of 40,940 machine-readable patterns with annotated names, including 40,724 automatically extracted names for cyclic substructures, 58 manually generated patterns for biologically relevant substructures, and 158 handcrafted patterns for functional groups. The web service has already been tested in a preliminary beta phase and will be maintained in the future, enabled by an easy-to-use bug report system that allows further improvement of the data set’s quality. The nonexistence of a machine-readable resource like this by IUPAC makes it mandatory to create it, as its usage can be diverse. While we utilize this collection to help scientists communicate, we hope to see further creative usage by others in the future. Especially AI methods and particularly those working with natural language might benefit from pattern collections like that of SmartChemist to comprehend and describe chemical substructures.

To summarize, we hope to contribute to improving communication about molecules in interdisciplinary groups like those found in many drug design teams. The importance of developing new drugs makes it imperative to improve any part of the drug design pipeline, including communication.

## Data Availability

The public SmartChemist web server is available at https://chemist.smarts.plus. The software is available under the BSD-3 license agreement and the patterns under the CC-BY-ND license agreement, both at https://github.com/torbengutermuth/SmartChemist.

## †

Global Discovery Chemistry, Biomedical Research, Novartis AG, 4056, Basel, Switzerland

## ‡

BASF SE, Ludwigshafen, 67056, Germany

## §

Bayer AG, Berlin, 13353, Germany

## References

[1] Baell J. B., Holloway G. A. (2010). New substructure
filters for removal of pan assay interference
compounds (PAINS) from screening libraries and for their exclusion
in bioassays. J. Med. Chem..

[2] Awale M., Hert J., Guasch L., Riniker S., Kramer C. (2021). The playbooks
of medicinal chemistry design moves. J. Chem.
Inf. Model..

[3] Ertl P. (2007). In silico
identification of bioisosteric functional groups. Curr. Opin. Drug Discovery Dev..

[4] Senior M. (2023). Fresh from
the biotech pipeline: fewer approvals, but biologics gain share. Nat. Biotechnol..

[5] Kim S., Chen J., Cheng T., Gindulyte A., He J., He S., Li Q., Shoemaker B. A., Thiessen P. A., Yu B. (2023). PubChem
2023 update. Nucleic Acids Res..

[6] Favre, H. A. ; Powell, W. H. International Union of Pure and Applied Chemistry. In Nomenclature of Organic Chemistry: Iupac Recommendations and Preferred Names 2013; Royal Society of Chemistry, 2024.

[7] Chalk, S. J. The IUPAC Gold Book Website, 2019.

[8] Lowe D. M., Corbett P. T., Murray-Rust P., Glen R. C. (2011). Chemical Name to
Structure: OPSIN, an Open Source Solution. J.
Chem. Inf. Model..

[9] Degtyarenko K., De Matos P., Ennis M., Hastings J., Zbinden M., McNaught A., Alcántara R., Darsow M., Guedj M., Ashburner M. (2007). ChEBI: a database
and ontology for chemical entities
of biological interest. Nucleic Acids Res..

[10] Daylight: SMARTS examples. https://www.daylight.com/dayhtml_tutorials/languages/smarts/smarts_examples.html#INTRO, (accessed 11 14, 2024).

[11] Daylight Chemical Information Systems, Inc. https://www.daylight.com/dayhtml/doc/theory/theory.smarts.html (accessed 02 12, 2025).

[12] Gandhi H.
A., White A. D. (2022). Explaining
molecular properties with natural language. ChemRxiv.

[13] Djoumbou
Feunang Y., Eisner R., Knox C., Chepelev L., Hastings J., Owen G., Fahy E., Steinbeck C., Subramanian S., Bolton E. (2016). ClassyFire: automated chemical classification
with a comprehensive, computable taxonomy. J.
Cheminf..

[14] Schomburg K., Ehrlich H.-C., Stierand K., Rarey M. (2011). Chemical pattern visualization
in 2D–the SMARTSviewer. J. Cheminf..

[15] Judson P. N., Ihlenfeldt W.-D., Patel H., Delannée V., Tarasova N., Nicklaus M. C. (2020). Adapting CHMTRN (CHeMistry TRaNslator)
for a new use. J. Chem. Inf. Model..

[16] RDKit: Open-Source Cheminformatics, version 2023.03.1. http://www.rdkit.org (accessed 11 14, 2024).

[17] Schmidt R., Ehmki E. S., Ohm F., Ehrlich H.-C., Mashychev A., Rarey M. (2019). Comparing molecular
patterns using the example of SMARTS: theory
and algorithms. J. Chem. Inf. Model..

[18] Ehmki E. S., Schmidt R., Ohm F., Rarey M. (2019). Comparing
molecular
patterns using the example of SMARTS: applications and filter collection
analysis. J. Chem. Inf. Model..

[19] https://github.com/django/django (accessed 02 24, 2025).

[20] https://github.com/benoitc/gunicorn (accessed 02 24, 2025).

[21] https://redis.io/(accessed 02 24, 2025).

[22] https://github.com/celery/celery (accessed 02 24, 2025).

[23] Mendez D., Gaulton A., Bento A. P., Chambers J., De Veij M., Félix E., Magariños M., Mosquera J. F., Mutowo P., Nowotka M. (2019). ChEMBL: towards direct deposition of bioassay data. Nucleic Acids Res..

[24] Hilbig M., Rarey M. (2015). MONA 2: A Light Cheminformatics Platform for Interactive Compound
Library Processing. J. Chem. Inf. Model..

[25] Tingle B. I., Tang K. G., Castanon M., Gutierrez J. J., Khurelbaatar M., Dandarchuluun C., Moroz Y. S., Irwin J. J. (2023). ZINC-22
A free multi-billion-scale database of tangible compounds for ligand
discovery. J. Chem. Inf. Model..

[26] Stierand K., Maaß P. C., Rarey M. (2006). Molecular complexes
at a glance:
automated generation of two-dimensional complex diagrams. Bioinformatics.

[27] Adasme M. F., Linnemann K. L., Bolz S. N., Kaiser F., Salentin S., Haupt V. J., Schroeder M. P. L. I.
P. (2021). PLIP 2021: expanding
the scope of the protein–ligand interaction profiler to DNA
and RNA. Nucleic Acids Res..

